# Malaria infection and anaemia in HIV-infected children in Mutengene, Southwest Cameroon: a cross sectional study

**DOI:** 10.1186/s12879-016-1853-z

**Published:** 2016-09-29

**Authors:** Ayukenchengamba Bate, Helen K. Kimbi, Emmaculate Lum, Leopold G. Lehman, Elias F. Onyoh, Lucy M. Ndip, Conica M. Njabi, Calvin Tonga, Godlove B.Wempnje, Roland N. Ndip, Pascal O. Bessong

**Affiliations:** 1 Department of Zoology and Animal Physiology, Faculty of Science, University of Buea, P.O. Box 63, Buea, SWR Cameroon; 2 Department of Medical Laboratory Sciences, Faculty of Health Sciences, University of Bamenda, Bamenda, P.O. Box 39, Bambili, NWR Cameroon; 3 Department of Biological Sciences, Higher Teachers’ Training College, University of Yaounde I, P.O. Box 47, Yaounde, Centre Region Cameroon; 4 Department of Animal Biology, Faculty of Science, University of Douala, P.O. Box 2701, Douala, Littoral Region Cameroon; 5 AIDS Care and Prevention Program, Cameroon Baptist Convention Health Services, Bamenda, Cameroon; 6 Institute of Epidemiology and Preventive Medicine, College of Public Health, National Taiwan University, Taipei, Taiwan; 7 Department of Microbiology and Parasitology, Faculty of Science, University of Buea, P.O. Box 63, Buea, SWR Cameroon; 8 Department of Biochemistry and Microbiology, Faculty of Science and Agriculture, University of Fort Hare, PMB X1314, Alice, 5700 South Africa; 9 HIV/AIDS and Global Health Research Programme, Department of Microbiology, University of Venda, Thohoyandou, 0950 South Africa

**Keywords:** Malaria parasite prevalence and density, HIV-infected children, Anaemia, Cameroon

## Abstract

**Background:**

Malaria is one of the leading causes of morbidity and mortality in children and HIV infection as well as other factors may worsen the situation. This study was aimed at determining the factors influencing malaria parasite prevalence and density as well as anaemia in HIV-infected children in Mutengene, Cameroon from November, 2012 to April, 2013.

**Methods:**

A semi-structured questionnaire was used to record information on socio-demographic factors and use of preventive measures by caregivers of HIV-infected children aged 1–15 years and of both sexes. Venous blood was collected; blood films were prepared and Giemsa-stained for parasite detection and speciation. Haemoglobin concentration was measured and the anaemic status determined. Data was analysed using Epi Info 7 software.

**Results:**

A total of 234 children were studied. The overall malaria parasite prevalence was 24.8 % (58) and was significantly higher (31.9 %, *P* = 0 .004) in females, those who did not implement any preventive measure at all (66.7 %, *P* = 0.03) and children who used antiretroviral therapy (ART) (28.6 %, *P* = 0.02) when compared with their respective counterparts. Geometric mean parasite density (GMPD) was significantly higher (3098.4, *P* = 0.02) in children who presented with fever, had CD4 T cells ≥500 cells/μL (491.3, *P* = 0.003) and those with moderate anaemia (1658.8, *P* = 0.03) than their respective counterparts. Although there was no significant difference, GMPD was however higher in males (549.0); those not on ART (635.0) and highest in children <5 years old (633.0) than their respective counterparts. The overall prevalence of anaemia was 49.6 % (116). The value was significantly highest (58.3 %, *P* = 0.01) in the 11–15 years age group; those with CD4 T cell level 200–499 (72.7 %, *P* = 0.001) and children with fever (85.7 %, *P* = 0.01).

**Conclusion:**

Implementation of proper and integrated malaria preventive measures as well as frequent monitoring of anaemia on prescription of ART could likely improve the health conditions of HIV-infected children thus avoiding malaria-related morbidity and mortality.

## Background

Malaria is a life threatening disease especially in children whose immune systems are not yet fully developed. The human immunodeficiency virus/acquired immunodeficiency syndrome (HIV/AIDS) infection further worsens the situation in children that are co-infected with both diseases. Unfortunately, HIV/AIDS geographically overlaps malaria infections in sub-Saharan Africa including Cameroon [1]. Approximately two-thirds of the global HIV/AIDS infected population lives in this area [2] and in 2013 an estimated 210,000 of infected persons were children [3]. More so, an estimated 163,000 malaria cases have been reported in children in sub-Saharan Africa [4]. Both infections are known to negatively impact each other. HIV-infected individuals are exposed to malaria infection on a daily basis [5] and have a higher risk of recurrent malaria episodes [6]. Malaria has also been associated with increased HIV replication in co-infected individuals [7]. Children are generally susceptible and vulnerable to malaria [8] and with HIV co-infection they may be at a higher risk of severe malaria infection. Such a co-infection may also retard the age-related acquisition of natural immunity to malaria in children [6] and lead to higher parasite densities thereby increasing the risk of anaemia [2, 5, 9, 10]. One of the major outcomes of HIV infection is also anaemia. There have been reports of high prevalence of childhood anaemia in the Mount Cameroon area [11] and Mutengene is one of the towns found in the area. Since malaria and HV/AIDS remain health concerns in this area, there is a need for constant monitoring of both diseases in all age groups.

Although there have been some reports on malaria in HIV patients in Cameroon [12–16], none of them has specifically focused on factors influencing the prevalence of malaria and anaemia in HIV-infected children. Such data remains very scanty in the country in general and the South West Region in particular. Baseline data on the prevalence of malaria and anaemia in this high risk group of patients is of utmost importance as this will help inform the design of interventions that can reduce the burden of both diseases. Against this background, this study was aimed at determining malaria parasite prevalence, density and anaemia in HIV-infected children attending the Care and Treatment Centre of the Mutengene Baptist Hospital, Southwest Cameroon.

## Methods

### Study area

The study was carried out in the Care and Treatment Centre of the Mutengene Baptist Hospital from November, 2012 to May, 2013. Mutengene is a semi-urban, road-junction town, located in the Mount Cameroon area of the South West Region, Cameroon. It is located at about 242 m above sea level, longitude 09° 18′ 29″ E and latitudes 04° 05′57″ N. It has an equatorial climate made up of a long rainy season that starts in mid-March and ends in mid-November. The dry season spans from mid-November to Mid-March. Weather records from the Cameroon Development Corporation show a temperature range, humidity and rainfall of 23 - 33 °C, 83.1 % and >4000 mm respectively per annum. Malaria transmission in the town is perennial and the entomological inoculation rate is reported as 36.76 bites/person/night [17].

### Study design and population

A cross-sectional study was carried out on HIV-infected children aged 1 to 15 years who came to the Care and Treatment Centre of the Mutengene Baptist Hospital (MBH). A total of 234 HIV-infected children from Mutengene and its environs in the outpatient department of MBH were enrolled in the study between November, 2012 and May, 2013. Before enrolment, a detailed explanation of the study and its potential benefits (using an information sheet) was given to parents/legal guardians of the children who were then invited to participate in the study. All the children who had been administered anti-malarials in the previous two weeks were excluded from the study.

A semi-structured questionnaire was administered to each parent/guardian and interviews were done in English and exceptionally in Pidgin English where necessary. The questionnaire sought for information on the age, sex, use of malaria preventive measures such as long lasting insecticide-treated nets (LLIN), insecticide residual spraying (IRS), presence/absence of stagnant water or bushes around the home and screening of windows and doors with wire mesh as well as use of and duration on ART. The questions were all closed ended questions. Patients were classified into different malaria prevention grades based on the number of mosquito preventive methods practised by the patient’s parent/guardian. They were classified into grades 0, I, II, and III^+^ if a patient/care-giver applied none, one, two or three and more preventive methods respectively.

### Ethical considerations

An ethical clearance was obtained from the Cameroon Baptist Convention (CBC) Institutional Review Board. Informed consent forms were given to the parents/legal guardians for reading and signature and an oral assent was gotten from the children. Only those whose care-givers accepted to allow them participate in the study by signing the written informed consent forms and who accepted to participate voluntarily were included in the study. All children below 1 year of age or those whose parents refused to allow them take part in the study or children who refused to participate in the study were excluded. All participants’ personal and health information were handled only by members of the research team and supporting health personnel. All patients’ data were held in total respect of medical confidentiality. Following the test results, participants that were diagnosed with malaria, severe anaemia and severe immunosuppression were immediately notified and referred to the clinician of the treatment centre for proper treatment and follow-up.

### Collection of blood samples

Before blood collection, the axillary temperature of each patient was measured. Fever was defined as temperature ≥ 37.5 °C.Venous blood was collected into well-labelled Ethylene Diamine Tetra Acetate (EDTA) tubes, and thick and thin blood films were prepared by the method of Cheesbrough [18] for the detection and speciation of malaria parasites respectively.

### Staining and microscopic examination of blood films

After fixing air-dried thin blood films with absolute methanol, both thick and thin blood films were stained with 10 % Giemsa for 30 min and examined under the ×100 (oil immersion) objective of an Olympus® BX 40 F light microscope (Olympus Optical Co. Ltd., Japan), for the detection and identification of malaria parasites, respectively, using the bench aids of Cheesbrough [18]. Slides were declared negative if no asexual parasites or gametocytes were found after examining 100 high-power fields. Slides were read by two independent parasitologists, and in the case of any disparity, they were read again by a third person. Malaria parasites were counted against 200 white blood cells (WBC) (or 500 WBCs in the case of very low parasitaemia) in thick blood films, and parasite density was expressed as number of parasites per microlitre (μL) of blood, assuming a WBC count of 8,000 leucocytes per μL of blood [18].

### Determination of haemoglobin concentration and anaemia

A Urit12 haemoglobinometer was used for the determination of haemoglobin (Hb) value. The Hb strips were placed on the haemoglobinometer and using a micropipette, approximately 15–20 μL of blood was put on the haemoglobinometer and the haemoglobin value was read in grams per decilitre (g/dL). Classification and severity of anaemia was done according to Sumbele et al. [19]. Haemoglobin values less than 11 g/dL were considered anaemic and anaemia severity was classified as follows: Hb values between 10.0-10.9 g/dL, 7.0–9.9 g/dL and < 7.0 g/dL were considered as mild, moderate and severe anaemia respectively.

### Determination of CD4+ T-lymphocyte count

This was done using the CyFlow® Counter (Partec GmbH, Münster, Germany). CD4 T-cell counts were categorised according to WHO [20] as low or advanced stage (<200/μl), moderate or chronic stage (200–499/μl) and high or asymptomatic stage (≥500/μl). Clinical stages were classified according to WHO criteria [20].

### Statistical analysis

The data was entered into Microsoft excel 2007. Data was analysed using Epi Info 7 software. Multiple logistic regression analyses was performed, Chi Square or Fischer’s exact tests were used to compare qualitative parameters. ANOVA (F), Kruskal-Wallis (H) or Mann–Whitney (U) tests were used to assess the differences between group means. Odds Ratios (OR) were calculated to compare the susceptibility of individuals or groups to different parameters. Multivariate analysis was done using multinomial logistic regression. Data was log transformed before the calculation of geometric mean parasite density (GMPD) was done for participants who were malaria-positive. Statistical significance was set at *P* < 0.05.

## Results

### Baseline characteristics of the study population

A total of 234 out of 239 HIV-infected children aged 1–15 years participated in the study. Five (2.1 %) children did not succumb to the blood collection procedure (although their parents had consented) and were therefore excluded from the study. The mean age of the children was 7.2 ± 3.6 years, 61.5 % of them had CD4 T cell counts ≥500 cells/μL of blood. There were more females (59 %, *n* = 138) than males (41.0 %, *n* = 96). A total of 182 (77.8 %) children were on ART while 52 (22.2 %) were not yet on ART. The overall malaria parasite prevalence was 24.8 % (*n* = 58) while that of anaemia was 49.6 % (*n* = 116).

### Malaria parasite prevalence and density with respect to age, sex, fever, CD4 T-cell counts and use of ART in the study population

In the multivariate analysis, young age (5–10 years; *P* = 0.031), sex (*P* = 0.007), clinical stage of the disease (*P* = 0.0016) and use of ART (*P* = 0.0001) were independent risk factors significantly associated with malaria parasite infection, as shown in Table 1. The highest malaria parasite prevalence was recorded in children aged 5–10 years (28.3 %) while the lowest was recorded in those aged 11–15 years (16.7 %). Although there was no significant difference, the highest GMPD (parasites/μL of blood) was recorded in children less than 5 years old (633.0 parasites/μL) and the lowest was recorded in those aged 5–10 years (234.0 parasites/μL).

**Table 1:** Factors influencing malaria parasite prevalence and density in a paediatric HIV infected population in Cameroon

Characteristic/category	Number examined	Number positive (%)	Bivariate analysis		Multivariate analysis		GMPD (range)	Statistic	*P*-value
			OR (95 % CI)	*P*-value^$^	OR (95 % CI)	*P*-value^£^			
Age Group (years)									
< 5	66	16 (24.2)	1.67 (0.62–4.12)	0.455	1.02 (0.28–3.71)	0.976	633.0 (80–24000)	H = 3.406	0.18
5–10	120	34 (28.3)	1.97 (0.84–4.67)	0.167	3.05 (1.11–8,42)	0.031*	234.0 (40–24000)		
11–15	48	8 (16.7)	Reference				463.3 (40–16000)		
Sex									
Males	96	14 (14.6)	Reference				549.0 (40–2400)	U = 218.0	0.10
Females	138	44 (31.9)	2.74 (1.40–5.36)	0.004*	3.08 (1.37–6.93)	0.007*	290.0 (40–24000)		
Fever									
Yes	14	4 (28.6)	1.23 (0.37–4.08)	0.985	1.38 (0.24–7.88)	0.716	3098.4 (400–24000)	U = 34.00	0.02*
No	220	54 (24.5)	Reference				287.3 (40–2400)		
CD4 T cell (cells/μl of blood)									
< 200	28	8 (28.6)	1.20 (0.49–2.96)	0.873	1.80 (0.55–5.85)	0.33	82.4 (40–360)	H = 11.598	0.003*
200–499	44	12 (27.3)	1.07 (0.37–3.06)	0.881	1.20 (0.46–3.13)	0.70	405.2 (80–16000)		
≥ 500	144	36 (25.0)	Reference				491.3 (40–24000)		
Clinical stages									
Stage 1	44	16 (36.4)	4.29 (1.28–14.4)	0,027*	10.38 (2.43–44.46)	0.0016*	191.2 (40–5040)	H = 14.36	0.002*
Stage 2	62	10 (16.1)	1.44 (0.42–5.00)	0.782	1.46 (0.36–5.87)	0.59	512.2 (40–5400)		
Stage 3	82	24 (29.3)	3.10 (0.99–9.77)	0.077	2.62 (0.76–9.09)	0.13	498.4 (80–2400)		
Stage 4	34	4 (11.8)	Reference				40.0 (40–40)		
Use of ART									
Yes	182	52 (28.6)	Reference				314.8 (40–24000)	U = 136.00	0.62
No	52	6 (11.5)	3.07 (1.24–7.61)	0.019*	0.194 (0.06–0.62)	0.0001*	635.0 (40–16000)		

*OR* Odds Ratio, *Statistically significant, *CI* Confidence Interval, ^$^:Estimated by Yates adjusted Chi-Square, ^£^:Estimated by Multiple Logistic Regression

In the bivariate model, malaria parasite prevalence was associated with sex with females (*χ*^2^ = 8.185, OR = 2.74, *P* = 0.004) being more likely to have malaria than males. On the contrary, GMPD was higher in males (549.0 parasites/μL) than females (290.0 parasites/μL), but the difference was insignificant.

Malaria parasite prevalence though not significant was higher (*χ*^2^ = 0.001, OR = 1.23, *P* = 0.99) in children presenting with fever (28.6 %, *n* = 4) than those who did not have fever (24.5 %, *n* = 54). GMPD was significantly higher (U = 34.00, *P* = 0.02) in children who presented with fever (3098.4, 95 % CI: 72.0–133263.4 parasites/μL) than in children who had no fever (287.3, 95 % CI: 61.3–1346.6 parasites/μL).

Malaria parasite prevalence was highest in children with CD4 T cell count <200 cells/μl (28.6 %, *n* = 8) and lowest in those with CD4 T cell count ≥ 500 cells/μL (25.0 %, *n* = 36), but there was no statistically significant difference (*χ*^2^ = 0.208, *P* = 0.90) as indicated in Table 1. The highest GMPD was recorded in those with CD4 T cells ≥500 cells/μL (491.3, 95 % CI: 252.0–957.8 parasites/μL) while the lowest was in those with a CD4 T cell count of <200 cells/μL (82.4, 95 % CI: 36.9–183.7 parasites/μL) and the difference was significant (H = 11.598, *P* = 0.003) as shown in Table 1.

Children on ART were 3 times more likely to have malaria parasites (*χ*^2^ = 5.414, OR = 3.07, *P* = 0.02) than those who were not on ART. Table 1 shows that GMPD was higher in children who were not on ART (635.0, 95 % CI: 37.2–10834.2 parasites/μL) than in those on therapy (314.8, 95 % CI: 189.3–523.5 parasites/μL) although the difference was not significant (U = 136.0, *P* = 0.62).

The highest malaria parasite prevalence was recorded in children at clinical stage 1 (36.4 %, *n* = 16) while the lowest was recorded in those at clinical stage 4 (11.8 %, *n* = 4) and the difference was significant (*χ*^2^ = 9.729, *P* = 0.02). The highest GMPD (512.2, 95 % CI: 231.3–1073.7 parasites/μL) was recorded in children at clinical stage 2 while the lowest (40, 95 % CI: 40–40 parasites/μL) was recorded in children at clinical stage 4 (Table 1) and the difference was significant (H = 14.36, *P* = 0.002).

### Malaria parasite prevalence and density in relation to preventive methods used by care givers of HIV-infected children

Malaria parasite prevalence in children using LLINs (22.9 %, *n* = 38) was lower than that in those not using LLINs (29.4 %, *n* = 20), but the difference was not significant (*χ*^2^ = 0.778, *P* = 0.38). GMPD was higher in those who did not use LLINs (408.2, 95 % CI: 136.2–1223.3 parasites/μL) than in those who did (306.7, 95 % CI: 176.8–531.9 parasites/μL), but the difference was not significant (H = 0.0271, *P* = 0.87).

**Fig. 1:**
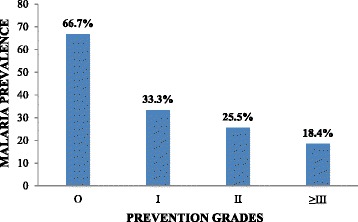
Malaria parasite prevalence in the different prevention grades

Although malaria parasite prevalence was higher in children who did not use IRS (25.6 %, *n* = 42), had bushes (29.3 %, *n* = 34) or stagnant water (36.0 %, *n* = 18) around their houses when compared with their respective counterparts the differences were however not significant. When all the mosquito preventive methods practised by care-givers of the participants were pulled together, the highest rate of infection was recorded in children whose care givers did not implement any mosquito preventive method at all, while the lowest was recorded in those whose care givers implemented at least three or more of the mosquito preventive methods (the use of bed nets, IRS, absence of stagnant water and bushes around the home) as shown in Fig. 1 and there was a significant difference between them (*χ*^2^ = 9.250, *P* = 0.03).

### Prevalence and density of malaria parasites in relation to anaemic status of HIV-infected children

The overall prevalence of anaemia in the study population was 49.6 % (116). Generally, 34.5 % (/40/116) of the anaemic cases were mild, 56.9 % (66/116) were moderate and 8.6 % (10/116) were severe. Although there was no statistically significant difference, the prevalence of malaria parasites was however higher in anaemia participants (25.9 %, *n* = 30) than in the non-anaemic ones (23.7 %, *n* = 28). The mean haemoglobin (±SD) concentration in the mild, moderate, and severe categories were 10.7 ± 0.51 g/dL, 9.2 ± 0.93 g/dL, and 7.2 ± 0.67 g/dL respectively, but there was no significant difference between them (*χ*^2^ = 5.321, *P* = 0.07). It was also observed that all the children with severe anaemia were negative for malaria (0.0 %, 0). GMPD was significantly higher (U = 58.0, *P* = 0.03) in those who were moderately anaemic (1658.8, 95 % CI: 459.6–5987.4 parasites/μL) when compared with their mildly anaemic counterparts (232.9, 95 % CI: 84.1–644.8 parasites/μL) as indicated in Table 2.

**Table 2:** Malaria parasite prevalence and density with respect to status of anaemia and severity in the study population

Characteristic/category	Number examined	Number positive for malaria (%)	OR (CI)	GMPD (range)
Anaemic status				
Non anaemic	118	28 (23.7)	Reference	164.5 (40–960)
Anaemic	116	30 (25.9)	1.12 (0.62–2.03)	663.6 (40–24000)
Level of significance		*χ* ^2^ = 0.051, *P* =0.82		U = 288.0, *P* = 0.04*
Anaemia severity				
Mild	40	14 (35.0)	Reference	232.9 (40–5040)
Moderate	66	16 (24.2)	0.59 (0.25–1.40)	1658.8 (40–24000)
Severe	10	0 (0.0)	---	0
Level of significance		*χ* ^2^ = 5.321, *P* = 0.07		U = 58.0, *P* = 0.03*

*Statistically significant, *CI* Confidence Interval, −-- Statistic that could not be computed

### Prevalence of anaemia in the study population with respect to age, sex, fever, use of ART, CD4 T cell counts and clinical staging

In multivariate analysis, young age (<5 years; *P* = 0.014), fever (*P* = 0.012) and CD4 T cell count (200–499; *P* = 0.00017) were independent risk factors significantly associated with anaemia. Children aged 11–15 years had the highest prevalence of anaemia (58.3 %, *n* = 28) while those aged <5 years had the lowest (33.5 %, *n* = 22); the difference was statistically significant only with bivariate analysis (*P* = 0.010) but not with the multivariate analysis. Anaemia was not associated with gender although female children had a higher prevalence of anaemia (53.6 %, *n* = 74) than the males (43.7 %, *n* = 42) (*χ*^2^ = 1.83, OR = 1.49, *P* = .18). Children who presented with fever were 6.69 times more likely to be anaemic (*χ*^2^ = 6.319, OR = 6.69, *P* = .01) than those who had no fever. Anaemia prevalence was significantly associated with the level of CD4 T cell count (*P* = 0.001) with the highest value recorded in children with 200–499 cells/μL (72.7 %, *n* = 32) and the lowest in those with ≥500 cells/μL (38.9 %, *n* = 56) as shown in Table 3. The prevalence of anaemia in children who were not on ART (57.7 %, *n* = 30) was slightly higher than in children who were on ART (47.3 %, *n* = 86) although the difference was not significant. Even though the WHO clinical staging was not significantly associated with anaemic status in children, it was observed that those in the clinical stage IV had the highest prevalence of anaemia (58.8 %, *n* = 20) while the least was recorded in those who were in clinical stage I of the HIV infection (40.9 %, *n* = 18).

**Table 3:** Factors influencing the prevalence of anaemia in the study population

Characteristic/category	Number examined	Number anaemic (%)	Bivariate analysis		Multivariate analysis	
			OR (95 % CI)	*p*-value^$^	OR (95 % CI)	*p*-value^£^
Age group (years)						
< 5	66	22 (33.3)	0.36 (0.17–0.77)	0.014*	0.68 (0.25–1.87)	0.45
5–10	120	66 (55.0)	0.87 (0.44–1.72)	0.825	0.74 (0.34–1.62)	0.45
11–15	48	28 (58.3)	Reference			
Sex						
Female	138	74 (53.6)	1.49 (0.88–2.51)	0.176	2.15 (1.11–4.15)	0.024*
Male	96	42 (43.7)	Reference			
Presence of fever						
No	220	104 (47.3)	Reference			
Yes	14	12 (85.7)	6.69 (1.46–30.60)	0.012*	3.65 (0.69–19.20)	0.13
CD4 T Cell category (cells/μl)						
< 200	28	16 (57.1)	2.10 (0.92–4.76)	0.114	1.99 (0.76–5.18)	0.16
200–499	44	32 (72.7)	4.19 (1.99–8.81)	0.00017*	3.79 (1.68–8.52)	0.0013*
≥ 500	144	56 (38.9)	Reference			
Clinical stages						
Stage 1	44	18 (40.9)	0.48 (0.12–1.20)	0.180	0.73 (0.24–2.17)	0.57
Stage 2	62	34 (54.8)	0.85 (0.36–1.98)	0.87	0.97 (0.35–2.64)	0.95
Stage 3	82	36 (43.9)	0.55 (0.24–1.23)	0.21	0.45 (0.18–1.15)	0.09
Stage 4	34	20 (58.8)	Reference			
Use of ART						
No	52	30 (57.7)	1.52 (0.82–2.84)	0.242	1.29 (0.60–1.29)	0.51
Yes	182	86 (47.3)	Reference			

*OR* Odds Ratio, *CI* Confidence Interval, ^$^: Estimated by Yates adjusted Chi-Square, ^£^: Estimated by Multiple Logistic Regression. *Statistically significant

## Discussion

Malaria and HIV co-infection in children is a condition of great public health significance as both infections have devastating effects on the health of children especially those less than 5 years old. Both infections lead to anaemia which can lead to serious consequences if not treated on time. The overall malaria parasite prevalence was 24.8 % in HIV-infected children. This value is similar to the 20.3 % malaria parasite prevalence reported in Uganda in both HIV-exposed and unexposed children [21]. The value is however less than that reported in some studies carried out in apparently healthy school children ≤15 years (33.8 % and 33.0 %) in the Mount Cameroon region [22, 23]. This discrepancy may be attributed to some state policies and strategies that have recently been implemented to fight HIV/AIDS and malaria in Cameroon. These include the free distribution of ART to HIV/AIDS patients in the country as the main control measure to improve their immune status and slow down the deteriorating effect of the virus. The number of care and treatment centres for HIV/AIDS distributing ART has increased in the last decade. Antimalarial strategies include the free distribution of LLINS, free treatment of malaria in children less than five years old and increased access to artermisinin-based combination therapies (ACTs) as well as malaria rapid diagnostic tests at subsidized rates. These measures have been relatively helpful especially in people living in urban areas. Similar measures contributed to reductions in malaria-related morbidity and mortality in HIV patients, particularly those living in urban settings in Mozambique [24]. Despite the involvement of the state and other development partners in the fight against HIV/AIDS in Cameroon, the coverage rate of antiretroviral treatment is still relatively low [25]. Unfortunately, malaria still remains a major cause of morbidity and mortality in Cameroon, especially among children and pregnant women, accounting for over 40 % hospital attendance [26].

Malaria parasite prevalence in this study was higher in children of younger age groups when compared with those of the older age groups. In malaria endemic areas acquired immunity to malaria is known to be both exposure- and age-dependent. Hence, older children might have developed some degree of immunity as a result of repeated malaria infections. This is in agreement with studies carried out in the Mount Cameroon region [22].

The study showed that females were significantly more infected with malaria parasites than males. This is in contrast with other studies [22, 27], which reported higher malaria prevalence rates in males. This may be due to the large proportion of females involved in this study. This could also be due to the fact that HIV is more common in females because of their biology and consequently leading to a lower level of immunity in them.

Children on ART were more likely to be infected with the malaria parasites than those who were not. This is in contrast with other studies which showed that the prevalence of malaria parasites was lower in patients who were on ART than in those who were not [28]. This discrepancy may be due to lack of adherence to therapy by the patients. Adherence to ART is a special issue in pediatrics because many of the drugs are not child friendly [29]. Adherence is important not only for individual patient responses to ART, but also because lack of adherence to prescribed regimens and sub-therapeutic levels of antiretroviral medications, particularly protease inhibitors, may enhance development of drug resistance and the likelihood of virologic failure. It may also be related to the relatively small sample size (22.2 %) of the patients who were not yet on ART in the study population. However, these findings were similar to one which showed that patients on ART had a slightly higher malaria prevalence than those who were not on ART [15]. On the other hand, GMPD was comparable in patients who were on ART and those who were not on ART. This finding is contrary to studies of other authors who reported that certain ARVs such as lopinavir/ritonavir can inhibit malaria parasite growth and reduce the incidence of malaria [28, 30, 31]. Although it was not investigated, it is worth noting that co-trimoxazole is generally prescribed for prophylaxis against opportunistic infections in HIV patients in treatment centres in Cameroon and it is also known to have anti-malaria properties [32].

When the vector control methods implemented by the participants in the study were all pulled together, it was observed that those who implemented at least three preventive methods had the least malaria prevalence, while those who did not implement any vector control method had the highest. This finding lends credence to the efficiency of possible integrated vector control methods against malaria infection. This needs to be encouraged in HIV-infected individuals.

The prevalence of anaemia in children in the study population was 49.6 %. This result was lower than values reported by Sumbele et al. [11] and Ruhinda et al. [33]. This may be due to the wide scale use of anti-malaria preventive measures that caused the decrease in the prevalence of malaria infections in these children and also the use of co-trimoxazole prescribed along with ART which has been associated with reduced anaemia in HIV infected children [34]. This confirms the fact that malaria is one of the major contributing factors to anaemia in children [35]. That probably also explains why GMPD was significantly higher in those who were anaemic as compared with their non-anaemic counterparts.

This study recorded a high prevalence of anaemia in the HIV-infected children who had lower CD4 T cell counts as well as those at clinical stage IV. This may be due to chronic inflammation by the HIV infection or it may be due to other opportunistic infections [36]. Sullivan et al. [37] and Mocroft et al. [38] associated anaemia to advancing HIV disease. The observed results could be due to the increasing viral burden which could cause anaemia through increased cytokine-mediated myelosuppression, and/or a higher burden of comorbidities. The use of ART in such patients usually leads to improvement in haemoglobin and other related haematological indices, even without specific additional treatment targeting anaemia [30]. It could possibly be linked to the kind of ART that the patients were taking as some of them have been reported to cause anaemia. Kimbi et al. [15] also reported high anaemia prevalence in patients who had CD4 T cells less than 200 cells/μL of blood in Limbe, Cameroon. Regardless of the mechanism, recognition of anaemia at initiation of ART could be useful to alert clinicians with regard to children requiring closer monitoring for possible treatment failure. It is also possible that anaemic children who did not respond adequately to ART might have been harbouring some drug resistant strains of HIV. Anaemia has been highlighted as an important independent prognostic factor in HIV-infected individuals. It is also thought to increase the risk of progression to AIDS and consequently death in HIV infected patients [36]. The adverse long term outcomes more likely reflect anaemia as a proxy of more advanced disease.

The oldest children (11–15 years) had the highest prevalence of anaemia while the youngest (<5 years) had the lowest. This contrasts with other studies carried out in southwest Cameroon [11, 22, 39] and Uganda [33]. Additionally, all children with severe anaemia were also malaria parasite negative. This difference could be attributed to the presence of the HIV infection and other possible opportunistic infections. It is worth noting that anaemia could have been caused by several factors including haemoglobinopathies, poor nutritional status, bacterial, viral and other parasitic infections like hookworm and *Entamoeba histolytica*. However, these were not investigated in this study.

Our findings also revealed a high prevalence of anaemia in children who presented with fever. This may be attributed to the high malaria prevalence and density observed in patients who presented with fever. The main mechanism of malaria anaemia pathogenesis involves the destruction of both infected and uninfected red blood cells [40] thus leading to a reduction in haemoglobin values.

The study is the first of its kind in Mutengene, Cameroon. Some limitations of this study include firstly the lack of data on a comparable group of HIV negative children in the same setting and this limits our ability to interpret the prevalence results precisely. A convenience sampling technique was used. This sampling was biased and the sample may not be a representative of the entire population. Self-reported information on mosquito preventive methods by participants was not verified and some of the information might have been biased. We were not able to get any information on viral loads as bouts of malaria are known to lead to higher viral loads. We did not have information on the nutritional status of the participants and this might have had an influence on anaemia in the patients. Our sample size was very large in the dry season as compared to the rainy season and this made seasonal comparison difficult.

### Conclusion

Proper implementation of malaria preventive measures and frequent monitoring of anaemia on prescription of ART are likely to improve on the health conditions of HIV-infected children thus reducing malaria-related morbidity and mortality in this vulnerable population.

## Abbreviations

AIDS: Acquired immunodeficiency syndrome
ART: Anti-retroviral therapy
ARV: Anti-retroviral
CBC: Cameroon Baptist Convention
CDC: Center for Disease Control
Hb: Haemoglobin
HIV: Human immunodeficiency virus
IRS: Insecticide residual spraying
ITNs: Insecticide treated nets
LLIN: Long lasting insecticide treated nets
WHO: World Health Organization
